# Outcomes of re-irradiation for brain recurrence after prophylactic or therapeutic whole-brain irradiation for small cell lung Cancer: a retrospective analysis

**DOI:** 10.1186/s13014-018-1205-9

**Published:** 2018-12-29

**Authors:** Ryoko Suzuki, Xiong Wei, Pamela K. Allen, James W. Welsh, James D. Cox, Ritsuko Komaki, Steven H. Lin

**Affiliations:** 1 Department of Radiation Oncology, Unit 97, The University of Texas MD Anderson Cancer Center, 1515 Holcombe Blvd, Houston, TX 77030-4009 USA; 2 Present address: Department of Radiation Oncology, Tokyo Medical and Dental University, 1 Chome-5-45 Yushima, Bunkyō, Tokyo, 113-8510 Japan

**Keywords:** CNS control, WBRT, Stereotactic radiosurgery, Survival

## Abstract

**Background:**

Small cell lung cancer (SCLC) can recur in the brain after whole-brain irradiation (WBI). We documented outcomes after treatment of such recurrences and sought predictors of local control and overall survival (OS).

**Materials and methods:**

Eighty-five patients with SCLC and brain recurrence after prophylactic or therapeutic WBI in 1998–2015 were identified and data were extracted from the medical records. Survival was estimated with the Kaplan-Meier method, and univariate and multivariate Cox proportional hazards modeling was used to identify factors associated with OS or further brain progression.

**Results:**

Brain recurrence was treated by stereotactic radiosurgery (SRS) in 33 patients (39%), repeat WBI in 14 (16%), chemotherapy-only in 16 (19%), and observation in 22 (26%). Median OS time after brain recurrence (OSrec) was 4.3 months for all patients; 6-month OSrec rates were 58% after SRS, 21% after repeat WBI, 50% after chemotherapy-only, and 5% after observation (*P* < 0.001). Inferior OSrec was associated with poor performance status (ECOG score ≥ 3) and uncontrolled extracranial disease. Superior OSrec was associated with receipt of ≥4 chemotherapy cycles before brain recurrence and receipt of chemotherapy, SRS, or repeat WBI afterward. Receipt of chemotherapy after brain recurrence correlated with brain progression.

**Conclusions:**

Some patients with brain recurrence after WBI for SCLC can survive for extended periods with appropriate intervention, especially those with adequate performance status or controlled extracranial disease.

## Background

Whole-brain irradiation (WBI) is an important component of treatment for small cell lung cancer (SCLC), whether given prophylactically or to treat known brain metastases. Because SCLC has a high propensity to seed in the brain, disease recurrence in the brain after WBI is not uncommon despite the radiosensitivity of the disease [1, 2]. Treatment strategies for brain recurrence after initial therapy for SCLC are limited but include surgical resection, stereotactic radiosurgery (SRS), repeat WBI, and chemotherapy.

In one autopsy series, 65% of patients had evidence of brain metastases at autopsy, with the probability of brain metastases increasing with survival time to 80% after 2 years [3]. Extended survival times from the use of modern radiotherapy techniques [4, 5] and the enhanced ability to detect small asymptomatic brain metastases [6] collectively increase the likelihood that more patients will manifest brain recurrence after initial WBI therapy for SCLC [6].

Repeat WBI for brain metastases from a variety of tumor types including lung cancer has been shown to be effective for symptom palliation [7–11]. A benefit from re-irradiating brain recurrences has also been found for patients with SCLC, although the median survival time after re-irradiation remains relatively short at 2 to 6 months [2, 12, 13]. In one study, patients with SCLC who developed brain metastases after prophylactic cranial irradiation (PCI) had effective symptom palliation from either repeat WBI or SRS [12]. Another series of patients with SCLC and brain relapse after WBI confirmed the feasibility of SRS as a salvage option [13, 14].

Nevertheless, information on how best to treat SCLC that has recurred in the brain after WBI is still sparse. Considering the high prevalence and depressing prognosis associated with brain recurrence, identifying reliable prognostic factors would be helpful for choosing among the various available treatment strategies.

In this single-institution retrospective study, we evaluated patients with SCLC who developed brain recurrence after WBI, with the goals of assessing outcomes and identifying prognostic factors that could influence survival and brain control.

## Methods

After approval of this study by the appropriate institutional review board, we identified 85 patients with SCLC and brain recurrence after WBI, given either with therapeutic or prophylactic intent, from 1998 through 2015 at a single tertiary cancer center. Management strategies for those brain recurrences (SRS, repeat WBI, chemotherapy, or observation) were extracted from the patients’ medical records, as was the following demographic and clinical information: factors at the initial diagnosis [sex, race, initial disease stage (limited vs. extensive), the presence and number of brain metastases at initial diagnosis (≤4 vs. > 4), intent of the first WBI (prophylactic vs. therapeutic), number of chemotherapy cycles before brain recurrences (< 4 vs. ≥4), and use and dose of thoracic radiation therapy (< 45 Gy vs. ≥45 Gy)]; factors at the brain recurrence [age, ECOG performance status (PS), symptoms and number of brain lesions (≤4 vs. > 4), the presence and status of extracranial disease (controlled vs. uncontrolled), and time from the initial WBI to the brain recurrence (< 6 months vs. ≥6 months)]; and factors after brain recurrence [treatment strategy (SRS, repeat WBI, chemotherapy only, or observation)]. Patients were typically given steroids, especially if they had symptoms, immediately upon diagnosis of brain recurrence. Patients with brain recurrence managed with observation were followed with best supportive care, including drugs such as steroids for symptom control.

The primary endpoint of this study was overall survival after brain recurrence (OSrec), which was defined as the time from the date of brain recurrence to the date of death. Local control in the brain after brain recurrence (BCrec), evaluated radiologically or clinically, was defined as the time from the date of brain recurrence to the date of detection of further brain recurrence. Patients lost to follow-up were censored at the date of the last follow-up. Progression was defined as lesions that persisted after SRS, repeat WBI, or chemotherapy or, in the observation group, as brain recurrence. The Kaplan-Meier method was used to assess survival and progression probabilities, and log-rank tests were used to compare Kaplan-Meier estimates of event rates between potential predictors. Univariate and multivariate Cox proportional hazards models were used to assess associations between factors and survival or further disease progression in the brain, and those factors with a *P* value < 0.1 were used to construct the final multivariate Cox model. All analyses were two-sided, and significance was set at *P* < 0.05. Statistical analyses were done with SPSS version 24 (SPSS, Chicago, IL, USA).

## Results

Patient characteristics, stratified by treatment for brain recurrence, are summarized in Table 1. Among all 85 patients with brain recurrence after WBI, 10 (12%) had poor PS (i.e., ECOG 3–4); 24 (28%) had had limited SCLC; and 33 (39%) had received WBI for prophylaxis, with the other 52 (61%) receiving WBI with therapeutic intent. All 85 patients had extracranial disease at brain recurrence, which was controlled in 29 (34%). The median interval between the first WBI and the brain recurrence was 6.0 months (range, 0.8–89.5 months).

**Table 1:** Patient characteristics by treatment received for brain recurrence

	All Patients (*n* = 85)	Group 1 (SRS) (*n* = 33)^a^	Group 2 (WBI) (*n* = 14)^b^	Group 3 (Chemo only) (*n* = 16)	Group 4 (Observation) (*n* = 22)
Age at brain recurrence, years					
< 63	42	16	6	10	10
≥63	43	17	8	6	12
ECOG performance status score at brain recurrence					
0–2	75	32	11	15	17
≥3	10	1	3	1	5
Sex					
Male	33	12	6	6	9
Female	52	21	8	10	13
Ethnicity					
White	69	23	11	15	20
Non-white	16	10	3	1	2
Initial disease stage					
Limited	24	12	3	2	7
Extensive	61	21	11	14	15
Brain metastases at initial diagnosis					
No	52	23	7	8	14
Yes	33	10	7	8	8
No. of brain lesions at initial diagnosis					
≤4	72	30	10	13	19
> 4	13	3	4	3	3
Intent of first brain irradiation					
Prophylactic	33	14	5	6	8
Therapeutic	52	19	9	10	14
≥4 chemo cycles before brain recurrence					
No	5	1	1	1	4
Yes	80	32	13	15	18
Thoracic radiation therapy dose, Gy					
< 45	28	6	7	7	8
≥45	57	27	7	9	14
No. of brain lesions at brain recurrence					
≤4	46	27	5	5	9
> 4	39	6	9	11	13
Symptoms from brain lesions at brain recurrence					
No	49	25	2	10	12
Yes	36	8	12	6	10
Extracranial disease at brain recurrence					
Controlled	29	15	5	5	4
Uncontrolled	56	18	9	11	18
Chemotherapy after brain recurrence					
No	55	20	13	0	22
Yes	30	13	1	16	0
Time from the end of first brain irradiation to brain recurrence, mo					
< 6	44	13	7	8	16
≥ 6	41	20	7	8	6

^a^Two patients received SRS with a Gamma knife after craniotomy

^b^One patient received local (not whole-brain) radiation to tumor site using conventional radiotherapy

*Abbreviations*: *SRS* stereotactic radiosurgery, *WBI* whole-brain irradiation, *ECOG* Eastern Cooperative Oncology Group

Brain recurrence was managed with SRS in 33 patients (39%), with repeat WBI in 14 (16%), with chemotherapy only in 16 (19%), and with observation (and best supportive care) in the remaining 22 patients (26%). Two of the patients in the SRS group had received SRS after craniotomy owing to the size of the lesion. One patient in the repeat WBI group had local fractionated radiotherapy rather than WBI. Of the 33 patients in the SRS group, 27 (82%) had ≤4 brain lesions at recurrence; by contrast, 9 patients (64%) in the repeat WBI group and 13 patients (59%) in the observation group had > 4 lesions. More than a third of patients in the SRS group also received additional chemotherapy, but only one patient in the repeat WBI group and no one in the observation group received further chemotherapy.

Nineteen patients in the SRS group (58%) had further brain failure, and 5 of those patients received a second SRS; 2 patients who received a second SRS had a third recurrence, which was treated with salvage WBI; and 1 was treated with salvage WBI. Thirteen patients in the chemotherapy-only group (81%) had further brain failure; 1 was treated with SRS, 1 was treated with SRS and had a second recurrence also treated with SRS, 2 were treated with SRS and had a second recurrence treated with salvage WBI, and 3 were treated with salvage WBI. Ten patients in the repeat-WBI group (71%) had further brain failure, of whom 1 had a craniotomy and the other 9 received best supportive care.

The median total dose of the first course of WBI was 30 Gy (range, 20–36 Gy), given in a median 3 Gy per fraction (range, 2–3 Gy). Most patients given repeat WBI received 20 Gy in ten 2-Gy fractions. For patients who received SRS, the prescribed dose was 12–20 Gy per lesion depending on their size and location.

**Fig. 1:**
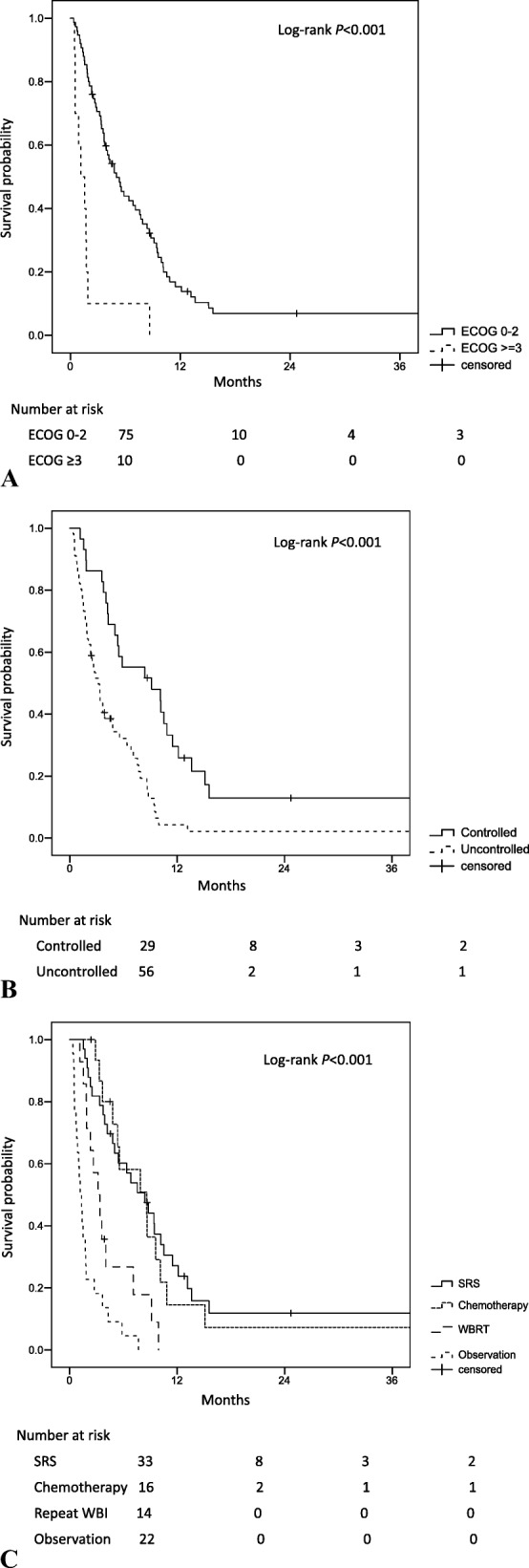
Kaplan-Meier plot of survival after brain failure after whole-brain irradiation for small cell lung cancer by (**a**) Eastern Cooperative Oncology Group performance status score (ECOG PS); (**b**) extracranial disease control; or (**c**) form of treatment for brain recurrence. Survival after brain failure was significantly better for patients with superior PS, extracranial disease control, and intervention than for those with poor PS, uncontrolled extracranial disease, and observation/best supportive care. SRS, stereotactic radiosurgery; WBI, whole-brain irradiation

The median OSrec time (i.e., survival after brain recurrence) was 4.3 months (95% confidence interval [CI] 2.7–5.9) for the whole group. Median OSrec time for those with ECOG PS 0–2 at recurrence was 5.0 months (95% CI 3.4–6.7) compared with 1.2 months (95% CI 0.1–2.2) for those with PS ≥3 (*P* < 0.001) (Fig. 1a). Median OSrec time for those with extracranial disease control at brain recurrence was 9.1 months (95% CI 1.9–16.4) compared with 3.2 months (95% CI 2.3–4.1) for those with uncontrolled extracranial disease (*P* < 0.001) (Fig. 1b). Rates of OSrec at 6 months were different according to type of treatment for the recurrence: 58% for the SRS group, 21% for the repeat WBI group, 50% for the chemotherapy-only group, and 5% for the observation group (*P* < 0.001) (Fig. 1c).

Further brain progression during follow-up was noted in 59 of the 85 patients. The median BCrec interval (i.e., brain control after brain recurrence) was 3.6 months (95% CI 2.2–5.1) for the entire group.

As for factors influencing OSrec, univariate analysis showed that worse OSrec was associated with poor PS (≥3) at recurrence, the presence of symptomatic brain lesions, and the presence of uncontrolled extracranial disease; better OSrec was associated with receipt of ≥4 chemotherapy cycles before brain recurrence, receipt of chemotherapy after brain recurrence, receipt of repeat WBI or SRS for brain recurrence, and an interval of ≥6 months from the end of the first WBI to the brain recurrence. Univariate analysis for factors associated with BCrec showed that worse BCrec was associated with poor PS (≥3), > 4 brain lesions at recurrence, symptomatic brain lesions, and uncontrolled extracranial disease; better BCrec was associated with receipt of repeat WBI or SRS for the brain recurrence. OSrec and BCrec did not differ for patients with limited- versus extensive-stage disease at initial diagnosis (Table 2).

**Table 2:** Univariate analysis of factors affecting survival and further brain control after recurrence in the brain after therapeutic or prophylactic irradiation

Characteristics	Survival after Brain Failure		Brain Control after Brain Failure	
	Hazard Ratio (95% CI)	*P* Value	Hazard Ratio (95% CI)	*P* Value
Age at brain recurrence, years				
< 63	1.00	0.757	1.00	0.633
≥63	0.931 (0.590–1.468)		0.881 (0.526–1.478)	
ECOG performance status score at brain recurrence				
0–2	1.00	< 0.001	1.00	0.007
≥3	4.66 (2.310–9.403)		2.776 (1.318–5.849)	
Sex				
Male	1.00	0.951	1.00	0.361
Female	0.985 (0.615–1.580)		1.285 (0.751–2.199)	
Ethnicity				
White	1.00	0.373	1.00	0.801
Non-white	0.762 (0.418–1.386)		0.921 (0.485–1.748)	
Initial disease stage				
Limited	1.00	0.955	1.00	0.129
Extensive	1.015 (0.605–1.703)		1.601 (0.872–2.940)	
Brain metastases at initial diagnosis				
No	1.00	0.656	1.00	0.519
Yes	1.112 (0.698–1.771)		1.19 (0.701–2.020)	
No. of brain lesions at initial diagnosis				
≤4	1.00	0.906	1.00	0.624
> 4	0.963 (0.518–1.793)		1.179 (0.610–2.278)	
Intent of first brain irradiation				
Prophylactic	1.00	0.910	1.00	0.853
Therapeutic	0.973 (0.606–1.564)		1.051 (0.621–1.778)	
≥4 chemo cycles before brain recurrence				
No	1.00	0.003	1.00	0.882
Yes	0.236 (0.091–0.614)		0.897 (0.215–3.752)	
Thoracic radiation therapy dose, Gy				
< 45	1.00	0.487	1.00	0.774
≥45	0.844 (0.524–1.361)		0.923 (0.532–1.600)	
No. of brain lesions at brain recurrence				
≤4	1.00	0.060	1.00	0.033
> 4	1.552 (0.981–2.454)		1.792 (1.049–3.063)	
Symptoms from brain metastases at brain recurrence				
No	1.00	0.003	1.00	0.043
Yes	2.041 (1.265–3.294)		1.718 (1.018–2.902)	
Extracranial disease at brain recurrence				
Controlled	1.00	< 0.001	1.00	0.012
Uncontrolled	2.683 (1.603–4.490)		2.084 (1.177–3.689)	
Chemotherapy after brain recurrence				
No	1.00	< 0.001	1.00	0.085
Yes	0.406 (0.248–0.663)		0.62 (0.360–1.069)	
WBI or G-knife for brain recurrence				
No	1.00	0.018	1.00	0.009
Yes	0.576 (0.365–0.908)		0.494 (0.290–0.842)	
Time from the end of first brain irradiation to brain recurrence, mo				
< 6	1.00	0.034	1.00	0.332
≥6	0.608 (0.384–0.963)		0.77 (0.460–1.291)	

*Abbreviations*: *ECOG* Eastern Cooperative Oncology Group, *WBI* whole-brain irradiation, *G-knife* Gamma knife therapy

In multivariate analysis, worse OSrec was associated with poor PS (≥3) at brain recurrence (hazard ratio [HR] 3.039, 95% CI 1.374–6.721, *P* = 0.006) and uncontrolled extracranial disease (HR 3.059, 95% CI 1.665–5.622, *P* < 0.001). Improved OSrec was associated with receipt of ≥4 chemotherapy cycles before brain recurrence (HR 0.351, 95% CI 0.126–0.975, *P* = 0.045); receipt of chemotherapy for brain recurrence (HR 0.174, 95% CI 0.094–0.324, *P* < 0.001); and receipt of repeat brain irradiation (with WBI or Gamma-knife) for brain recurrence (HR 0.367; 95% CI 0.205–0.656; *P* < 0.001). As for BCrec, receipt of chemotherapy after brain recurrence was borderline significant in univariate analysis for BCrec (*P* = 0.085) but became significant in multivariate analysis after adjustment for other variables that were significant at *P* < 0.1 in univariate analysis (HR 0.457, 95% CI 0.250–0.832, *P* = 0.01), indicating that receipt of chemotherapy after brain recurrence may be associated with better BCrec. Uncontrolled extracranial disease and receipt of repeat brain irradiation (with WBI or Gamma-knife) for brain recurrence were marginally significant in multivariate analysis of BCrec (*P* = 0.055 and *P* = 0.050). No other factors were found to be associated with BCrec in multivariate analysis (Table 3).

**Table 3:** Multivariate analysis of factors affecting survival and further brain control after recurrence in the brain after therapeutic or prophylactic irradiation

Characteristics	Survival after Brain Failure		Brain Control after Brain Failure	
	Hazard Ratio (95% CI)	*P* Value	Hazard Ratio (95% CI)	*P* Value
ECOG performance status score at brain recurrence				
0–2	1.00	0.006	1.00	0.103
≥3	3.039 (1.374–6.721)		1.955 (0.874–4.372)	
≥4 chemo cycles before brain recurrence				
No	1.00	0.045	–	–
Yes	0.351 (0.126–0.975)			–
No. of brain lesions at brain recurrence				
≤4	1.00	0.116	1.00	0.142
> 4	1.534 (0.900–2.615)		1.572 (0.859–2.875)	
Symptom from brain metastases at brain recurrence				
No	1.00	0.376	1.00	0.499
Yes	1.310 (0.721–2.380)		1.233 (0.672–2.260)	
Extracranial disease at brain recurrence				
Controlled	1.00	< 0.001	1.00	0.055
Uncontrolled	3.059 (1.665–5.622)		1.855 (0.987–3.600)	
Chemotherapy after brain recurrence				
No	1.00	< 0.001	1.00	0.01
Yes	0.174 (0.094–0.324)		0.457 (0.250–0.832)	
WBI or G-knife for brain recurrence				
No	1.00	0.001	1.00	0.050
Yes	0.367 (0.205–0.656)		0.533 (0.283–1.001)	
Time from first brain irradiation to brain recurrence, mo				
< 6	1.00	0.153	–	–
≥6	0.679 (0.400–1.154)		–	

*Abbreviations*: *ECOG* Eastern Cooperative Oncology Group, *WBI* whole-brain irradiation, *G-knife* Gamma knife therapy

Ten of the 85 patients in our study lived for more than 1 year after the first brain recurrence (Table 4). Notably, 8 of those 10 patients did not have liver or bone involvement at initial diagnosis. Moreover, Patients 5 and 7, who received temozolomide (without re-irradiation) for the initial recurrence later underwent SRS for a subsequent brain recurrence. Therefore all 10 of these patients had received SRS, whether for the initial or subsequent brain recurrence.

**Table 4:** Characteristics of the ten patients who lived more than 1 year after brain recurrence

	Patient 1	Patient 2	Patient 3	Patient 4	Patient 5	Patient 6	Patient 7	Patient 8	Patient 9	Patient 10
OSrec, mo.	45.8	13.2	13.6	12.1	57.8	15.6	15.1	12.8	24.7	41.5
Age at brain recurrence, years	62	61	71	63	57	64	69	63	64	65
ECOG PS score at brain recurrence	0	0	1	1	0	1	1	1	0	1
Sex	Male	Female	Female	Female	Female	Female	Female	Male	Male	Female
Initial disease stage (limited or extensive)	Extensive	Extensive	Extensive	Extensive	Extensive	Extensive	Extensive	Extensive	Limited	Limited
Extrathoracic organs involved at initial diagnosis	Brain, adrenal	Bone	Liver	Thyroid	Brain	Brain	Brain	None ^a^	None	None
No. of brain lesions at brain recurrence (1, 2–4 or ≥ 5)	1	2–4	2–4	2–4	≥5	1	≥5	1	1	1
Symptoms from brain lesions at brain recurrence (no or yes)	No	No	No	No	No	No	No	No	No	Yes
Extracranial disease at brain recurrence	Controlled	Uncontrolled	Controlled	Controlled	Controlled	Controlled	Controlled	Controlled	Controlled	Uncontrolled
Time from the end of first brain irradiation to brain recurrence, mo.	8.3	11.3	2.5	4.6	13.4	16.2	7.5	5.1	8.9	5.1
Treatment received for brain lesion after brain recurrence	SRS	SRS	SRS	SRS	Temozolo-mide	SRS (after surgery)	Temozolo-mide	SRS	SRS	SRS
Chemotherapy after brain recurrence (no or yes)	Yes	Yes	Yes	No	Yes	No	Yes	No	No	Yes
Additional brain failure after initial brain recurrence after WBI (no or yes)	Yes	No	Yes	Yes	Yes	Yes	Yes	Yes	Yes	No
Salvage therapy for additional brain failure after initial brain recurrence after WBI	Chemo	–	Chemo	SRS, repeat WBI afterwards	SRS	SRS	SRS	Observation	Observation	–

^a^Separate tumor nodule in contralateral lobe

*Abbreviations*: *OSrec* overall survival time after brain recurrence, *ECOG PS* Eastern Cooperative Oncology Group performance status, *SRS* stereotactic radiosurgery, *WBI* whole-brain irradiation

## Discussion

Available options for patients with disease recurrence in the brain after WBI for SCLC are limited, but our findings suggest that some patients can expect better survival when those recurrences are treated with therapeutic intent by using SRS, repeat WBI, or chemotherapy, especially patients with adequate PS or controlled extracranial disease.

Despite the radiosensitivity of SCLC, brain recurrence after WBI is not uncommon [1, 2]. In one meta-analysis of 7 trials comparing PCI with no PCI for patients with limited-stage SCLC, 27% of all patients had disease recurrence in the brain [1]. Another retrospective series revealed brain recurrence in 15% of patients with SCLC after WBI; all of those recurrences were treated with either repeat WBI (12%) or SRS (3%) [2].

In the current study, we found that both PS and extracranial disease control were important contributors to survival after brain recurrence. Although these results were not entirely consistent with those of other studies (perhaps because of patient heterogeneity), some other studies also found these factors to be important for survival in such cases. Among several studies of prognostic factors and outcomes after re-irradiation with WBI for various malignancies including lung cancer [7–11], one showed that repeat WBI relieved symptoms in 71% of treated patients, and even though the median survival time after re-irradiation was short at 4 months, the 1-year survival rate for patients with no extracranial metastases was better at 14% versus only 4% for those with extracranial metastases [7]. A similar study also found better prognosis among patients with stable extracranial disease at re-irradiation [8]. In addition to extracranial disease control, high Karnofsky performance score (KPS) [9–11], the absence of severe symptoms [10], and longer interval between the two courses of WBI [11] have also been correlated with improved survival in other studies.

A few studies have also examined the feasibility of SRS for brain metastases after WBI for various cancers, including lung [15–17]. In one such study, salvage SRS produced a median survival time of 8.4 months overall, and was longer for patients with a single brain metastasis than for those with multiple brain metastases (12.0 vs. 7.9 months) [15]. Another study found that high KPS, controlled extracranial metastases, and a good response to re-irradiation all had positive effects on survival [16]. The importance of KPS was also confirmed in another study, along with neurologic function [17].

Although available data are limited, we did find a few studies that addressed treatment for brain recurrences after WBI in SCLC. One study reported a benefit from brain retreatment with WBI or SRS among patients with SCLC and post-PCI brain metastases; in that study, 40% of symptomatic patients achieved symptom palliation after re-irradiation, and the median survival times were 3 months after repeat-WBI and 5 months after SRS. That study further showed that patients with higher KPS (≥50) had significantly improved survival after re-irradiation, a finding that is comparable to ours [12]. The importance of PS in survival was also confirmed in a study that used salvage SRS after WBI; other factors found to be significant were tumor volume and the interval between SCLC diagnosis and the appearance of brain metastases [14]. Another retrospective study assessing the efficacy of salvage SRS after WBI for SCLC showed that although the median OS time for all patients was 5.9 months, 24% of patients survived for at least 12 months, and patients with no evidence of extracranial disease had significantly better survival than did patients with stable or progressive extracranial disease [13].

Our findings also showed that patients who received ≥4 cycles of chemotherapy *before* brain recurrence had better survival, perhaps because of a lower systemic disease burden. We also found that patients who received chemotherapy *after* brain recurrence had better survival and better brain control than those who did not. Although several new chemotherapy agents have been evaluated in phase II trials for second-line treatment of SCLC, to date they have not resulted in changes to standard practice, which is currently platinum plus etoposide for first-line therapy and topotecan for second-line therapy [18]. Further improvements in systemic therapy, such as targeted agents and immune checkpoint inhibitors, are eagerly awaited.

We found that all 10 of the patients who lived for more than 1 year after brain recurrence received SRS, whether for initial or subsequent brain recurrence, a finding that highlights the importance of local treatment to improve outcome [19, 20].

The graded prognostic assessment (GPA) is advocated as a reliable and useful tool for estimating survival and for selecting appropriate treatment strategies for patients with brain metastases [21]. The original GPA score included age, KPS, number of brain lesions, and the presence or absence of extracranial metastases and has been validated for its prognostic potential in lung cancer [22, 23]. Although we also identified PS and extracranial disease control as significant prognostic factors, we could not calculate GPA in our patient population because they not only had recurrent disease but they also tended to have a more extensive systemic disease burden than the patients included in the development of the GPA analysis.

This study had some limitations that should be considered in interpreting our results, chief among them being its retrospective nature, broad patient heterogeneity, and relatively small sample size. We cannot rule out the possibility that our findings could reflect selection bias. Thus our results should be considered exploratory and ideally should be validated in a larger, prospective cooperative group study.

## Conclusions

The prognosis for patients with SCLC that recurs in the brain after WBI is still rather dismal, but survival in some cases, particularly those with adequate PS and controlled extracranial disease, can be extended if the recurrence is managed therapeutically.

## Abbreviations

BCrec: Brain control after brain recurrence
ECOG: Eastern Cooperative Oncology Group
GPA: Graded prognostic assessment
KPS: Karnofsky performance score
OS: Overall survival
OSrec: Overall survival after brain recurrence
PCI: Prophylactic cranial irradiation
PS: Performance status
SCLC: Small cell lung cancer
SRS: Stereotactic radiosurgery
WBI: Whole-brain irradiation
